# Development of an innovative double-chamber syringe for intravenous therapeutics and flushing: Nurses’ involvement through a human-centred approach

**DOI:** 10.1371/journal.pone.0235087

**Published:** 2020-06-25

**Authors:** Pedro Parreira, Liliana B. Sousa, Inês A. Marques, Paulo Costa, Sara Cortez, Filipa Carneiro, Arménio Cruz, Anabela Salgueiro-Oliveira

**Affiliations:** 1 Health Sciences Research Unit: Nursing (UICISA:E), Nursing School of Coimbra (ESEnfC), Coimbra, Portugal; 2 Health Sciences Research Unit: Nursing (UICISA:E), Nursing School of Coimbra (ESEnfC), Coimbra, Portugal; 3 Coimbra Institute for Clinical and Biomedical Research (iCBR) area of CIMAGO, CNC.IBILI, Faculty of Medicine, University of Coimbra, Coimbra, Portugal; 4 Health Sciences Research Unit: Nursing (UICISA:E), Nursing School of Coimbra (ESEnfC), Coimbra, Portugal; 5 Muroplás - Plastic Engineering Industry, Muro, Portugal; 6 PIEP Innovation in Polymer Engineering, Guimarães, Portugal; 7 Health Sciences Research Unit: Nursing (UICISA:E), Nursing School of Coimbra (ESEnfC), Coimbra, Portugal; 8 Health Sciences Research Unit: Nursing (UICISA:E), Nursing School of Coimbra (ESEnfC), Coimbra, Portugal; Universiteit Twente, NETHERLANDS

## Abstract

**Background:**

In nursing practice, flushing the catheters pre and post-drug administration is considered an important clinical procedure to prevent complications, and requires the use of several syringes to comply with international standards of care. We envisioned an innovative double-chamber syringe that enables the filling and administration of both solutions. Following current international recommendations, the development of new medical devices should integrate Health Technology Assessment. The Human-centred design is usually used for that assessment purposes, as a method that actively include end-users in the devices development process.

**Method:**

Application of the Human-Centred Design through the involvement of nurses in the initial stages of the device development in order to accomplish the initial stages of Technology Readiness Level. A multi-method approach was used, including literature/guidelines review, focus groups with end-users and expert panels.

**Results:**

The involvement of nurses enabled the definition of user requirements and contexts of use, as well as the evaluation of design solutions and prototypes in order to accomplish with usability and ergonomic features of the medical device.

**Conclusions:**

Significant contributions were made regarding the final design solution of this innovative double-chamber syringe.

## Introduction

The insertion of a peripheral intravenous catheter (PIVC) is the most frequent invasive procedure performed in nursing clinical practice, which enables the administration of nutrients, fluids, drugs and blood products directly on the bloodstream [1,2]. However, there is a wide range of mechanical, chemical or infectious complications which can impact the patient’s safety [3–7]. Thus, the prevention of such complications has been the main focus of scientific research worldwide. International guidelines highlight some care precautions during PIVC insertion (e.g. hand hygiene, aseptic technique, catheter size, anatomical insertion site, proper securements) and maintenance (e.g. daily site inspection, flushing), highlighting the need for recurrent professional training [8–10]. PIVC flush is one of the most important factors in preventing catheter malfunction. By maintaining catheter patency, this procedure can prevent recurrent complications such as occlusion, phlebitis, and infection [9]. In fact, the theoretical purpose of flushing is to maintain catheter patency by preventing internal luminal occlusion, reducing build-up of blood or other products on the device internal surface and preventing interaction of fluids or drugs [11–15].

International standards of care in this thematic scope recommend a minimum flush volume equal to twice the internal volume of the catheter system (catheter, extension set and/or needleless injection system) [9]. PIVC are traditionally flushed before and after drugs administration [9, 16]. The flushing practice implies the daily assessment of the device patency, as well as a minimum pre and post flush after each single drug administration, using a pulsatile technique [9]. Despite clear recommendations, flushing practices appear to vary widely, especially when focusing on the correct solutions, frequency, volumes, and techniques [17–19]. Traditionally, this process requires the use of two or three separate syringes per each drug administration: an initial syringe, to assess PIVC patency and vein integrity; the second syringe, to deliver the prescribed drug; and a third syringe (or the first again), to perform a final PIVC flush.

To decrease costs and contamination risks, prefilled flush syringes have become available in the international market [20]. Despite the known advantages, the use of prefilled flush syringes in clinical settings is minimal [19]. Alternatively, double-chamber syringes have been developed to enable the sequential administration of intravenous drugs followed by the administration of a flushing solution, reducing contamination risks, costs and procedural time. Some of these syringes have not been widely adopted because most of the designs only allow the use of the flushing chamber once (before or after the drug administration). Other dual-chamber systems have been developed that allows drug combination or reconstitution and subsequent administration [21]. The need to use at least two syringes to comply with international standards of care remains. To address this challenge, the “Duo Syringe” project was initiated with the main purpose of developing an innovative medical device (MD), a double-chamber syringe, that enables the filling and administration of drugs and flush solution (before and after drug delivery).

### Legal requirements for the development of new MDs

The regulatory system for MDs in the EU is specified in the recently reviewed European Council Regulation (EU) 2017/745 of the European Parliament and of the Council of 5 April 2017 on MD, amending Directive 2001/83/EC, Regulation (EC) No178/2002 and Regulation (EC) No1223/2009 and repealing Council Directives 90/385/EEC and 93/42/EEC [22]. To ensure the uniform application of such EU directives, MEDDEV consensus statements and interpretative documents were developed. These EU directives require greater transparency and compromise solutions that will benefit both patients and MD manufacturers.

MDs released in the marketed in their earlier development stages are more likely to increase the risk of harm [23]. These concerns led to the development of international recommendations that highlight the need to integrate the Health Technology Assessment (HTA) core assumptions in the development of new MDs [24–25] in order to prevent those risk of harm.

HTA processes involve the determination of significant information regarding clinical, economic, social, and ethical value of health technologies (either pharmaceuticals or MDs). Although HTA guidelines are widely accepted and well defined for the evaluation of pharmaceuticals, its generic application to non-drugs technologies such as MDs has not been equally consensual. Internationally, the regulatory processes are substantially less stringent for MD than for drugs [26–28], with outstanding differences between them regarding the nature of the clinical evidence base, the nature of the device’s technology, the incremental innovation, the learning curve effects, and the organizational impact [29]. Currently, several challenges remain in the assessment of MDs and making clinical and cost-effectiveness decisions [30–32]. There is a need for better linkage between licensing policies and innovative HTA procedures [26], in order to increase patient safety, particularly in the European Union (EU) [27,33], as emphasized by the European Clinical Research Infrastructure Network (ECRIN) [34].

According to HTA Core Model classification (EUnetHTA) [35], both drugs and MDs reports should consistently consider safety, effectiveness, and economic evidence. Facing this, research involving the final users and investigate their needs may contribute for product development may contribute with a more objective approach in earlier stages of MD development, considering an iterative process [36]. In fact, following good design principles and involving users in the earlier phases of the MD development process (considering the users’ involvement) decreases the need for design changes. This would not only save time and money, but also diminish the frustration for both developers and users. Considering the EU directives, the manufacturer is required to “reduce, as far as possible, the risk of use error due to ergonomic features of the device and the environment in which the device is intended to be used” [22]. Similarly, the International Organization for Standardization (ISO) emphasizes the need to evaluate “the extent to which a user can use a product to achieve specific goals with effectiveness, efficiency and satisfaction in a specific context” (IEC ISO 62366:2015) [37]. According to these regulatory requirements, Human-centred design (HCD) appears as a method that involves the user in the development process, in order to ensure that MD meets their needs and competences, but also to improve safety, satisfaction, effectiveness and efficiency, while reducing product recalls and modifications [38–41]. In order to achieve the international standards (ISO 13407:1999, ISO 14155:2011; ISO 14971:2012) [42–44], the HCD method defines four phases in the development of MDs: (i) identify the user and specify the context of use; (ii) specify the user requirements; (iii) produce design solutions; and (iv) evaluate design solutions against requirements. The main goal of this iterative method is to increase the involvement of end-users throughout the design and development process of new MDs in order to enhance its effectiveness, efficiency, and satisfaction in a specific context of use [37,38].

### Study objectives

The main purpose of this study is to describe and report the initial steps in the development of a new MD (the Duo Syringe). According to the European requirements previously outlined, an HCD approach was used with the involvement of the end-user of this double-chamber syringe (nurses). Specifically, we intend to: (i) explore the conceptual idea about this new device, establishing the users and their requirements, as well as the contexts of use; (ii) obtain enough data regarding such aspects in order to develop design solutions; (iii) assess those design solutions against the initial requirements; and (iv) select the better design solution and determine any necessary modification in order to improve the MD.

## Method

### Design and procedures

**Fig 1:**
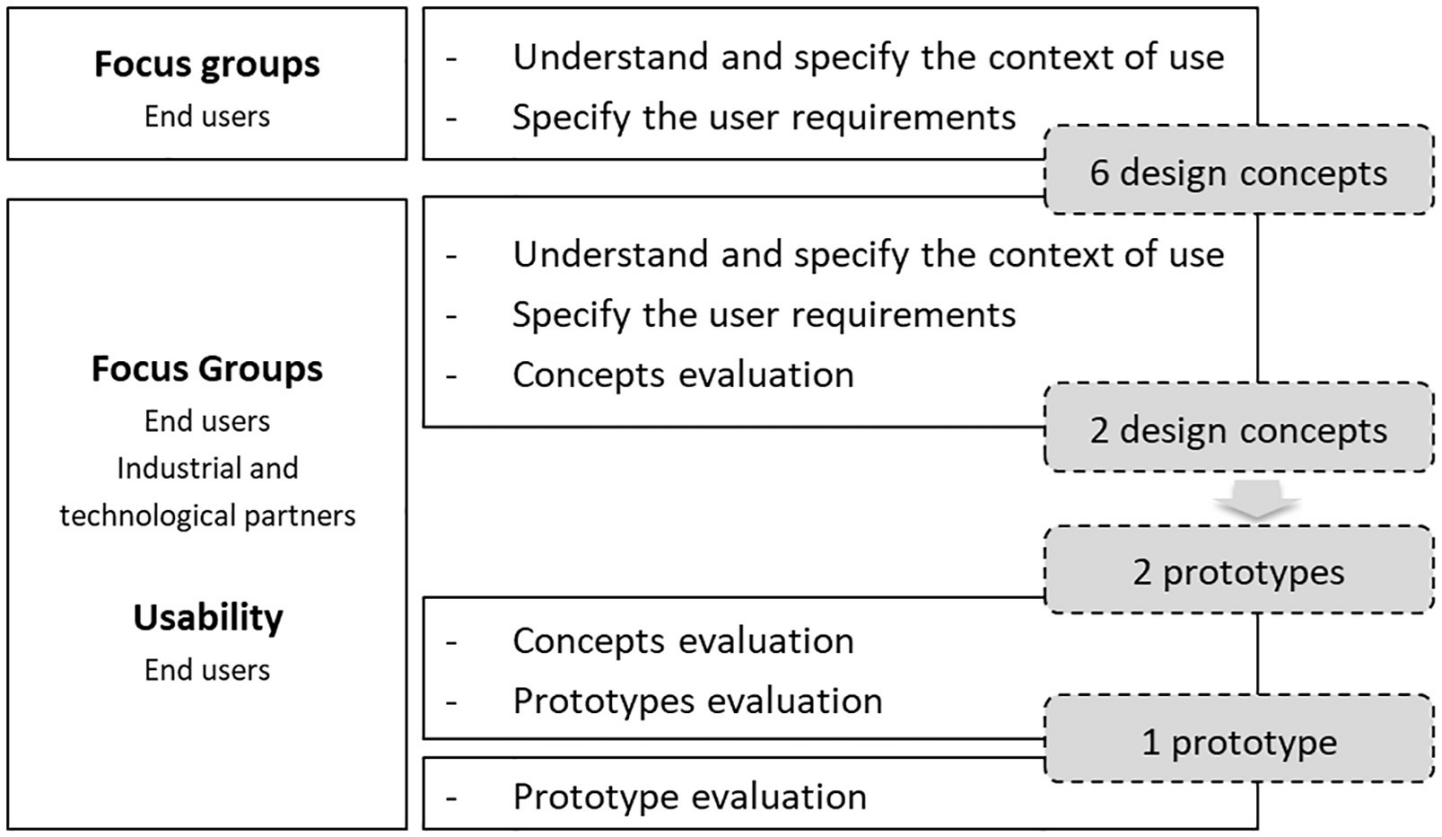
Flowchart of the study.

This study was reviewed and approved (No. P608-8/2019) by the ethics Committee of the Health Sciences Research Unit: Nursing (UICISA: E) of the nursing School of Coimbra (ESEnfC). The HCD model requirements were used to define a specific multi-method approach to implement along the initial stage of the Duo Syringe’s development [45,46]. According to this, several steps were implemented during the Duo Syringe’s development process (Fig 1).

#### Contexts of use and user requirement

An initial literature review focused on the international guidelines about peripheral intravenous catheterization and infusion therapy standards of care was performed, not only to identify the clinical practices, current care gaps, and end-users (both health care professionals and the patients), but also to define preliminary functional requirements and characteristics for the device (e.g. dimensions of the syringe’s body and plunger, volume required for each chamber). Focus groups with nurses were done to specify the users’ needs and requirements, alongside with the determination of the contexts of use, as well as any potential barriers to a safer and effective use of this double-chamber syringe (Table 2). All the sessions were audio-recorded and then transcribed for qualitative analysis. An additional panel of experts was conducted with the project’s academic, industrial and technological partners in order to provide them with the necessary information for designing the device concept.

#### Design solutions for the device

Six design concepts were developed through software NX (version 11.0; Siemens Product Lifecycle Management Software Inc.) by the technological partner (see appendix for design images and semi-functional prototypes photos).

#### Assessment of the design solutions

The Duo Syringe concepts were analysed by the project’s panel of experts composed by academia, industrial and technological partners, who had previously elaborated a matrix with essential assessment criteria and its ponderation values. The semi-functional prototypes of the two solutions with the higher scores were assessed by the nurses in new focus groups (Table 1) with nurses (Table 2). The prototypes were evaluated with respect to their ergonomic and usability characteristics, potential applicability barriers (their causes and consequences), as well as suggestions for device improvement. The final design solution was then selected by the project’s research team (academic, technological and industrial partners) based on the qualitative (focus groups) and quantitative results (assessment matrix).

**Table 1:** Focus groups guidelines.

Steps	Duration (minutes)	Purposes
I. Introduction and consent	5	Presentation of the research project main objective; Obtain informed consent; Communicate about the group main purposes.
II. Concept(s)	15	Presentation of the MDs’ concept(s) (with the support of a 3D video).
III. Semi-functional prototype(s)	15	Presentation of the MD prototypes.
IV. Discussion	20	Ensure that the discussion focus is the MDs’ characteristics. Ask for potential modifications to improve the MDs.
V. Sample characterization and ending	5	Show appreciation for participation and obtain information to characterize the group.

**Table 2:** Focus Groups: Users’ characterization (N = 16).

		N	%	M	SD	Min.-Max.
**Sex**	Male	5	31.25			
	Female	11	68.75			
**Age (years)**				39.25	10.096	25–55
**Education**	Bachelors’ degree	3	18.75			
	Post-graduate/Specialty	4	25.00			
	Master degree	9	56.25			
**Professional time (months)**				195.56	120.434	36–372
**Department**	Research unit	3	18.75			
	Management support	1	6.25			
	Operating room	2	12.50			
	Surgery/Cardiothoracic surgery	3	18.75			
	Intensive care	2	12.50			
	Oncological Gastroenterology	1	6.25			
	Orthopaedics	2	12.50			
	Pneumology	1	6.25			
	Burn unit	1	6.25			
	Public institutions	13	81.25			
	Other (Teaching/Research)	3	18.75			
**Time at the current professional unit (months)**				135.25	124.509	6–372

M–Mean; SD–Standard Deviation; Min.–Minimum; Max.–Maximum.

### Materials

Supplementary materials were used in the focus groups with users: (i) focus groups guidelines (according to the structure presented in Table 1); (ii) an informed consent document, which gave a brief description of the research study, stated the main purpose of the groups and the voluntary nature of participation; (iii) demographic questionnaire to characterize the nurses’ age, gender, educational and professional data.

### Sample

The focus groups with users involved the participation of 16 nurses, with distinct education levels and whose clinical practice settings differ. The sample characterization is presented in Table 2.

### Data analysis

Data collected included individual characteristics and demographics. Data were entered and analysed using the *Statistical Package for the Social Sciences–version 22*.*0* (SPSS 22.0, SPSS Inc., Chicago, IL, USA). Means, standard deviations, frequencies, and percentages were used as descriptive statistics. For qualitative analysis (focus groups), the content analysis technique was used [47], considering pre-defined categories about usability and functional requirements. Other categories were also measured (*a posteriori*), according to the content of the users’ groups.

## Results

### Contexts of use and user requirements

The main advantages for this double-chamber syringe recognised by the focus groups participants (nurses) were related to the importance of the flushing practice, for example regarding the patency assessment, as well as to prevent drugs interaction or contamination. Also, this new device will bridge some major reasons for the non-adherence to flushing procedure, such as time constraints or limited human resources. In fact, these syringes will also be an important contribution to the time constraints, which is important due to the limited human resources in the units, as stated by the participants. Other general advantages pointed by the nurses were the reduced number of manipulations, with associated improvements in terms of safety for patients and professionals (reducing infections and obstructions). The nurses identified several clinical and care contexts in which this double-chamber syringe might be used, such as emergency departments, angiography suits, implanted or semi-implanted catheters, chemotherapy, and operating rooms.

Several functional requirements were considered, regarding the plunger and syringe body dimensions (as close as possible to the syringes on the market), the device design, the volume for each chamber (10 ml for the drugs), injection speed for the solutions (ensure the requirements for the bolus or intravenous administration), intended sequences for the chamber filling and administration of both solutions (flush solution for patency evaluation, administration of the drugs, and flush solution again). Considering the several clinical contexts of their professional activity, the majority of the participants in the focus groups agreed upon a 10 ml volume for each chamber. Concerning this, a few participants stated that 10 ml for drugs could be insufficient in a particular oncological context (such as cytostatic or some antibiotics). The drug administration should respect the maxim pressure established for 10 ml syringes of 25 psi. The syringe should enable the verification of the catheter patency through the administration of the flush solution, the subsequent drugs delivery and the final flushing of the catheter using the remaining flush solution.

### Design solutions for the device

The industrial and technological partners developed six concepts based on user requirements and contexts of use previously identified by the academic partner, but also attending to legal standards required to the industrial process of a class I non-invasive MD with metrics. All the concepts developed allow for the filling of two independent chambers, one with a flush solution and the other with an intravenous drug. One of the concepts (third concept) requires a syringe switch in order to alternate access to each of the two chambers. In the administration phase, all concepts enable the pre-flushing or patency assessment, the drug delivery, and subsequent flushing of the catheter. One exception is the second developed concept which only allows the use of each chamber once, undermining attempts of a procedure following all the steps recommended in international standards of care.

### Assessment of the design solutions

Several criteria were determined by the academia, industrial and technological partners, namely: (i) dimensions for both the syringe’s body and plunger; (ii) number of needed manipulations; (iii) possibility of error in the fluids administration sequence; (iv) number of components; (v) product complexity and assembly; (vi) costs; (vii) ease of filling the syringe chambers; (viii) ease of administration; (ix) potential vascular trauma to the patient. These requirements were rated by each partner regarding their importance to the selection of the best concept (Tables 3–5), and the mean ponderation value for each variable was calculated (Table 6).

**Table 3:** Academic partner: Ponderation table for the variables to be assessed.

	(i)	(ii)	(iii)	(iv)	(v)	(vi)	(vii)	(viii)	(ix)	total
(i) dimensions		0.5	0	1	1	1	0.5	0.5	0	4.5
(ii) number of manipulations	0.5		0.5	1	1	1	0.5	0.5	0	5.0
(iii) possibility of error	1	0.5		1	1	1	1	1	0.5	7.0
(iv) number of components	0	0	0		1	0.5	0	0	0	1.5
(v) productive complexity and assembly	0	0	0	0		0	0	0	0	0
(vi) costs	0	0	0	0.5	1		0	0	0	1.5
(vii) ease of filling the syringe chambers	0.5	0.5	0	1	1	1		0.5	0	4.5
(viii) ease of administration	0.5	0.5	0	1	1	1	0.5		0	4.5
(ix) potential vascular trauma	1	1	0.5	1	1	1	1	1		7.5

0-less important; 0.5-equally important; 1-more important

**Table 4:** Technological partner: Ponderation table for the variables to be assessed.

	(i)	(ii)	(iii)	(iv)	(v)	(vi)	(vii)	(viii)	(ix)	total
(i) dimensions		0	0	1	0.5	0	0.5	0.5	0	2.5
(ii) number of manipulations	1		0	1	1	0.5	0.5	0.5	0	4.5
(iii) possibility of error	1	1		1	0.5	0	1	1	1	6.5
(iv) number of components	0	0	0		0.5	0	0	0	0	0.5
(v) productive complexity and assembly	0.5	0	0.5	0.5		0	0	0	0	1.5
(vi) costs	1	0.5	1	1	1		0.5	0	0.5	5.5
(vii) ease of filling the syringe chambers	0.5	0.5	0	1	1	0.5		0.5	0	4.0
(viii) ease of administration	0.5	0.5	0	1	1	1	0.5		0	4.5
(ix) potential vascular trauma	1	1	0	1	1	0.5	1	1		6.5

0-less important; 0.5-equally important; 1-more important

**Table 5:** Industrial partner: Ponderation table for the variables to be assessed.

	(i)	(ii)	(iii)	(iv)	(v)	(vi)	(vii)	(viii)	(ix)	total
(i) dimensions		0.5	1	1	0	0	1	1	0.5	5
(ii) number of manipulations	0.5		0.5	0.5	0	0	0	0	0	1.5
(iii) possibility of error	0	0.5		1	0	0	0	0	0	1.5
(iv) number of components	0	0.5	0		0	0	0	0	0	0.5
(v) productive complexity and assembly	1	1	1	1		0	1	1	1	7.0
(vi) costs	1	1	1	1	1		1	1	1	8.0
(vii) ease of filling the syringe chambers	0	1	1	1	0	0		0	0	3.0
(viii) ease of administration	0	1	1	1	0	0	1		0	4.0
(ix) potential vascular trauma	0.5	1	1	1	0	0	1	1		5.5

0-less important; 0.5-equally important; 1-more important

**Table 6:** Concepts assessment: Ponderation table for the variables to be assessed.

	Academic	Technological	Industrial	Mean ponderation value
(i) dimensions	4.5	2.5	5	4.0
(ii) number of manipulations	5.0	4.5	1.5	3.7
(iii) possibility of error	7.0	6.5	1.5	5.0
(iv) number of components	1.5	0.5	0.5	0.8
(v) productive complexity and assembly	0	1.5	7.0	2.8
(vi) costs	1.5	5.5	8.0	5.2
(vii) ease of filling the syringe chambers	4.5	4.0	3.0	3.8
(viii) ease of administration	4.5	4.5	4.0	4.2
(ix) potential vascular trauma	7.5	6.5	5.5	6.5

The potential vascular trauma induced to the patient was identified by the experts as the most important dimension to consider in the selection of the better design, followed by the manufacturing costs and the possibility of error.

After defining a common matrix, the panel assessed all the Duo Syringe concepts (Table 7). During this stage, the second concept was excluded by the experts because it did not enable the pre-flushing of the PIVC.

**Table 7:** Concepts assessment: Final scores.

	(i)	(ii)	(iii)	(iv)	(v)	(vi)	(vii)	(viii)	(ix)	total
1 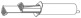	5 (20)	4 (14.8)	5 (25)	5 (4)	5 (14)	5 (26)	5 (19)	4 (16.8)	4 (26)	**165.6**
2 EXCLUDED										
3 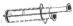	5 (20)	1 (3.7)	3 (15)	3 (2.4)	2 (5.6)	2 (10.4)	4 (15.2)	4 (16.8)	2 (13)	102.1
4 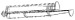	2 (8)	5 (18.5)	4 (20)	3 (2.4)	4 (11.2)	4 (20.8)	5 (19)	5 (21)	5 (32.5)	**153.4**
5 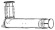	2 (8)	2 (7.4)	5 (25)	5 (4)	1 (2.8)	1 (5.2)	4 (15.2)	3 (12.6)	3 (19.5)	99.7
6 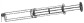	1 (4)	4 (14.8)	2 (10)	5 (4)	4 (11.2)	4 (20.8)	5 (19)	4 (16.8)	4 (26)	126.6

Concept 2 was excluded because it did not enable the pre-flushing; Likert scale for concept assessment: 0-terrible; 1-very weak; 2-weak; 3-sufficient; 4-good; 5-excellent; (in parenthesis are presented the corrected values according to ponderation values achieved for each evaluation criteria).

The two concepts with better scores (the first and fourth concepts) were then subjected to rapid prototyping processes and discussed by nurses during a focus group. Nurses selected the first prototype as the final one and gave feedback to improve it in terms of usability/ergonomic characteristics of the syringe. The nurses’ feedback focused mainly on the syringe’s plunger, body, and chambers.

Regarding the plunger, and considering the semi-functional prototype manipulation by participants, no annotations were made regarding the plunger course. Nevertheless, minor modifications related to the dimension and colour of both plungers were annotated. Specifically, the nurses suggested a higher support base for the plungers, maintaining a circular shape like in traditional syringes. In order to differentiate the drug chamber and the flushing solution chamber, it was suggested using different colours in each chambers (e.g. blue or green for the drug chamber plunger). In fact, the differentiation of the chambers was a major concern for the participants in a way that enhanced clinical practice safety. Some participants also emphasized the need to ensure that one chamber is only used for the flushing solution (in a way that both chambers cannot be used for two drugs). Another suggestion for such differentiation was through different scale marking (a larger scale on the flushing chamber and a smaller scale for the drugs chamber), although this was not considered significant by all the participants. All the participants identified the black colour for the scale on the syringe body as their preference (mirroring the design of traditional syringes).

During the analysis of the semi-functional prototype, the dimensions of the syringe body were considered suitable by the participants. It was suggested the enhancement of the support flaps for the index and medium fingers (particularly important for nurses with bigger hands). A major concern emerged regarding the centred *Luer lock* system, because of the risk of mechanically induced vascular trauma to the patient (in connecting the *Luer lock* adapter to the catheter). Facing this, a substantial number of participants suggest the classical *Luer slip* system on the border to reduce this risk. The steps for intravenous drug preparation and administration using the new syringe was another aspect discussed in the focus groups. The filling sequence of each chamber should be standardized: the flush solution chamber should be the first to be charged, and then the drug chamber (to avoid contamination). Accordingly, the participants suggested a stopper system on the first chamber (flush solution) in order to facilitate the charge of the second chamber (drug).

The final design solution (design solution number 1) was chosen by nurses in final focus groups, mainly due to its simplicity both during the preparation and administration phases (easier, reduce errors in the chambers charging). Also, those design solutions achieved the higher value in the quantitative assessment from the academic, technological and industrial partners.

## Discussion

The development of MDs should be an iterative process, where the assessment is an important step. The HCD methodological approach [48] involves the end-user in the MD development process, with several purposes: (i) to obtain successful products that improve patient safety and satisfaction; (ii) ensuring that the device meets the users’ needs and competences; (iii) increase device effectiveness and efficiency; and (iii) reducing product recalls and modifications [38,39]. According to this, and in order to accomplish with national and international regulations, we applied the HCD method to the development of a new device: a double-chamber syringe for intravenous therapeutics that enables drug administration and the pre and post PIVC flush in a sequential logic, improving health professionals’ adherence to current international standards of care. Specifically, the HCD’ four phases were used in initial stages of product development (identification of users, contexts of use and user requirements), but also for produce and evaluate design solutions.

End-user involvement in the MD development process has been considered necessary to obtain successful products, i.e. not only to define the user requirements and contexts of use, but also to evaluate design solutions and prototypes. In fact, to produce viable and well-adjusted prototypes it is necessary to verify if the user requirements have been met and that the device adheres to basic usability and human factors/ergonomics principles. Prototypes play an important role in the development of new MDs allowing for the assessment of shape and form before manufacturing [49]. Thus, identifying and recruiting representative end-users is essential to ensure a reliable data set [50,51].

For this double-chamber syringe, focus groups with primary end-users (nurses) were considered in several development stages. These focus groups resulted in the identification of potential barriers to the safe and effective use of the double-chamber syringe, through the careful analysis of the semi-functional prototype. The main contributions made by the participants were related to the syringe’s plunger (e.g. with different colours to visually differentiate the drug and flushing chambers), body (e.g. enhancement of the support flaps for the index and medium fingers on the syringe body) and chambers (e.g. the filling and administration sequences). Moreover, significant recommendations were done, for example, regarding the need to ensure that the two chambers were used for their intended purpose (i.e. one chamber for the intravenous drugs and the other chamber for the flushing solution). In fact, the notion of *intended purpose* is an imperative aspect to consider in product development particularly from the manufacturers’ perspective [52].

Recent SWOT analysis of the European MD industry emphasized the lack of strong connections between industries and academia [53]. Although *stakeholder input* in the MD development process is considered an overwhelming challenge because it requires more time and resources [27], this collaboration is strongly recommended [40]. Our study enables a better comprehension about the multidisciplinary skills as well as perspectives and contributions of the partners involved in the development of a double-chamber syringe. In fact, the expert panels composed by elements of the academic, industrial, and technological sectors provided decisive contributions to the product development on a design and prototype level, which resulted in the collection of essential data for the manufacturers [49]. During the product development process, the need to redesign a product requires these stakeholders’ *input* in order to: (i) improve usability and aesthetics, reduce human errors, increase safety and efficiency, improve patient outcomes and satisfaction (from the perspective of the users); as well as (ii) add new functionalities or change materials, reduce the number of components and associated costs, limit the need for *ad hoc* modifications, improve manufacturability and/or mechanical properties (from the industry or technological partners’ perspective) [39,54].

Future research on this double-chamber syringe includes the pre-clinical validation of the functional prototype in simulation context (labs) through usability tests with end-users (nurses), as well as its clinical validation in real clinical settings (hospitals) [55].

## Conclusions

The application of the HCD method during the double-chamber syringe design and development processes brings a simple yet robust structure for the concept outline and prototype evaluation. A detailed description of the activities carried on in each phase was conducted, applying an ergonomics and human factors approach to this real example of MD development. This flexible and useful method enables the selection, development, and refinement of the final design solution for this double-chamber syringe, constituting a valid approach to close the gap between the final solution and the end-users’ needs, where efficacy and safety are highlighted. Adopting these formal methods in the decision-making process effectively assist both industrial and technological partners to take a more integrated, objective and reflective approach into a device development, which should result in successful and high-quality products.

## Supporting information

S1 File (PDF)

S2 File (PDF)

## References

[1] Chopra V.; Flanders S.A.; Saint S.; Woller S.C.; O’Grady N.P.; Safdar N.; … Michigan Appropriateness Guide for Intravenous Catheters (MAGIC) Panel. The Michigan Appropriateness Guide for Intravenous Catheters (MAGIC): results from a multis-pecialty panel using the RAND/UCLA appropriateness method. *Ann Intern Med* 2015, 163(6 Suppl), S1–S40. 10.7326/M15-0744 26369828

[2] Corrigan A. Infusion nursing as a specialty In *Infusion nursing*: *An evidence-based approach*, 3^rd^ ed; Alexander M., Corrigan A., Gorski L., Hankins J., Perucca R., Eds.; Saunders/Elsevier: St Louis, US, 2010, pp. 1–9.

[3] Abdul-Hak C.K.; Barros A.F. The incidence of phlebitis in a Medical Clinical Unit. *Texto Contexto Enferm* 2014, 23(3), 633–8. 10.1590/0104-07072014000900013

[4] Braga L.M.; Parreira P.M.; Oliveira A.S.S.; Mónico L.S.M.; Arreguy-Sena C.; Henriques M.A. Phlebitis and infiltration: Vascular trauma associated with the peripheral venous catheter. *Revista Latino-Americana de Enfermagem* 2018, 26: e3002 10.1590/1518-8345.2377.3002 29791668 PMC5969824

[5] Danski M.T.R.; Oliveira G.L.R.; Johann D.A.; Pedrolo E.; Vayego S.A. Incidence of local complications in peripheral venous catheters and associated risk factors. *Acta Paul Enferm* 2015, 28(6), 517–23.

[6] Rojas-Sánchez L.Z.; Parra D.I.; Camargo-Figuera F.A. Incidence and factors associated with the development of phlebitis: results of a pilot cohort study. *Rev Enf Ref* 2015, Série IV(4), 61–7.

[7] Salgueiro-Oliveira A.; Parreira P.; Veiga P. Incidence of phlebitis in patients with peripheral intravenous catheters: the influence of some risk factors. *Aust J Adv Nurs* 2012, 30(2), 32–9.

[8] Capdevila J.A.; Guembe M.; Barerán J.; Alarcón A.; Bouza E.; Fariñas M.C.; et al on behalf the SEICAV, SEMI, SEQ, and SECTCV Societies. 2016 Expert consensus document on prevention, diagnosis and treatment of short-term peripheral venous catheter-related infections in adults. *Cir Cardiov* 2016, 23(4), 192–198. 10.1016/j.circv.2016.06.001 27580009

[9] Infusion Nurses Society. Infusion therapy standards of practice. *Journal of Infusion Nursing* 2016, 39(1S), S1–160.

[10] Loveday HP.; Wilson J.A.; Pratt R.J.; Golsorkhi M; Tingle A.; Bak A; et al Epic3: National evidence-based guidelines for preventing healthcare-associated infections in NHS hospital in England. *Journal of Hospital Infection* 2014, 86 S1, S1–S70. 10.1016/S0195-6701(13)60012-2 24330862 PMC7114876

[11] Bishop L.; Dougherty L.; Bodenham A.; Mansi J.; Crowe P.; Kibbler C.; et al Guidelines on the insertion and management of central venous access devices in adults. *International Journal of Laboratory Hematology* 2007, 29, 261–278. 10.1111/j.1751-553X.2007.00931.x 17617077

[12] Guiffant G.; Durussel J.J.; Merckx J.; Flaud P.; Vigier J.P.; Mousset P. Flushing of intravascular access devices (IVADs)—Efficacy of pulsed and continuous infusions. *The Journal of Vascular Access* 2012 13(1), 75–78. 10.5301/JVA.2011.8487 21748725

[13] Ngo A.; Murphy S. A theory-based intervention to improve nurses’ knowledge, self-efficacy, and skills to reduce PICC occlusion. *Journal of Infusion Nursing* 2005, 28(3), 173–181. 10.1097/00129804-200505000-00005 15912072

[14] Royon L.; Durussel J.J.; Merckx J.; Flaud P.; Vigier J.P.; Guiffant G. The fouling and cleaning of venous catheters: A possible optimization of the process using intermittent flushing. *Chemical Engineering Research and Design* 2012, 90(6), 803–807. 10.1016/j.cherd.2011.10.004

[15] Vigier J.P.; Merckx J.; Coquin J.Y.; Flaud P.; Guiffant G. The use of a hydrodynamic bench for experimental simulation of flushing venous catheters: Impact on the technique. *ITBM-RBM* 2005, 26(2), 147–149. 10.1016/j.rbmret.2005.03.001

[16] Royal College of Nursing. *Standards for infusion therapy*, 4^th^ ed; Royal College of Nursing: London, 2016. 10.12968/bjon.2018.27.2.S12 29368570

[17] Parreira P.; Marques I.; Santos-Costa P.; Sousa L.B.; Braga L, Apóstolo J.; et al Peripheral venous catheter flushing: a scoping review protocol. *Revista de Enfermagem Referência* 2020, 5(1):e19066.

[18] Goossens GA. Flushing and locking of venous catheters: Available evidence and evidence deficit. *Nursing Research and Practice* 2015, 985686. 10.1155/2015/985686 26075094 PMC4446496

[19] Keogh S.; Flynn J.; Marsh N.; Higgins N.; Davies K.; Rickard C. Nursing and midwifery practice for maintenance of vascular access device patency. A cross-sectional survey. *International Journal of Nursing Studies* 2015, 52, 1678–1685. 10.1016/j.ijnurstu.2015.07.001 26206327

[20] Keogh S.; Marsh N.; Higgins N.; Davies K.; Rickard C. A time and motion study of peripheral venous catheter flushing practice using manually prepared and prefilled flush syringes. *Journal of Infusion Nursing* 2014, 37(2), 96–101. 10.1097/NAN.0000000000000024 24583939

[21] Werk T.; Ludwig I.S.; Luemkemann J.; Mahler H-C.; Huwyler J.; Hafner M. Technology, applications, and process challenges of dual chamber systems. *Journal of Pharmaceutical Sciences* 2016, 105, 4–9. 10.1016/j.xphs.2015.11.025 26852837

[22] European Commission. Regulation (EU) 2017/745 of the European Parliament and of the Council of 5 April 2017 on medical devices; Author: Brussels, Belgium, 2017.

[23] Hwang T.J.; Sokolov E.; Franklin J.M.; Kesselheim A.S. Comparison of rates of safety issues and reporting of trial outcomes for medical devices approved in the European Union and United States: Cohort study. *BMJ* 2016, 353: i3323. 10.1136/bmj.i3323 PMC4925918 27352914

[24] Clinical Excellence Commission. The Device Usability Handbook–An introductory resource for NSW health employees. Clinical Excellence Commission: Sydney, Australia, 2017.

[25] Polisena J.; Castaldo R.; Ciani O.; Federici C.; Borsci S.; Ritrovato M.; et al Health Technology Assessment methods guidelines for medical devices: How can we address the gaps? The International Federation of Medical and Biological Engineering perspective. *International Journal of Technology Assessment* 2018, 34:3, 276–289. 10.1017/S0266462318000314 29909792

[26] Ciani O.; Wilcher B.; Giessen A.V.; Taylor R. S. Linking the regulatory and reimbursement processes for medical devices: The need for integrated assessments. *Health Economics* 2017, 26(Suppl. 1), 13–29. 10.1002/hec.3479 28139087

[27] Fuchs S.; Olberg B.; Panteli D.; Perleth M.; Busse R. HTA of medical devices: Challenges and ideas for future from a European perspective. *Health Policy* 2017, 121, 215–229. 10.1016/j.healthpol.2016.08.010 27751533

[28] Tarricone R.; Boscolo P.R.; Armeni P. What type of clinical evidence is needed to assess medical devices? *European Respiratory Review* 2016, 25, 259–265. 10.1183/16000617.0016-2016 27581825 PMC9487219

[29] Tarricone R.; Callea G.; Ogorevc M.; Rupel V.P. Improving the methods for the economic evaluation of medical devices. *Health Economics* 2017, 26(Suppl. 1), 70–92. 10.1002/hec.3471 28139085

[30] Kramer D.B.; Yeh R.W. Practical improvements for medical device evaluation. *JAMA* 2017, 318(4), 332–334. 10.1001/jama.2017.8976 28742887 PMC6150452

[31] Schnell-Inderst P.; Mayer J.; Lauterberg J.; Hunger T.; Arvandi M.; Conrads-Frank A.N.; et al Health technology assessment of medical devices: What is different? An overview of three European projects. *Z*. *Evid*. *Fortbild*. *Qual*. *Gesundh*. *Wesen* 2015, 109, 309–318. 10.1016/j.zefq.2015.06.011 26354131

[32] Sedrakyan A.; Campbell B.; Merino J.G; Kuntz R.; Hirst A.; McCulloc P. IDEAL-D: A rational framework for evaluating and regulating the use of medical devices. *BMJ* 2016, 353: i2372. 10.1136/bmj.i2372 27283585

[33] Tarricone R.; Torbica A.; Drummond M. Challenges in the assessment of medical devices: The MedTecHTA project. *Health Economics* 2017, 26(Suppl. 1), 5–12. 10.1002/hec.3469 28139084

[34] Neugebauer E.A.M.; Rath A.; Antoine S.L.; Eikermann M.; Seidel D.; Koenen C.; et al Specific barriers to the conduct of randomized clinical trials on medical devices. *Trials* 2017, 18: 427 10.1186/s13063-017-2168-0 28903769 PMC5597993

[35] EUnetHTA Joint action European Network for Health Technology Assessment Joint Action on HTA 2012–2015: HTA core model for rapid relative effectiveness. EUnetHTA: Diemen, Netherlands, 2015.

[36] Martin J. L.; Barnett J. Integrating the results of user research into medical device development: Insights from a case study. *BMC Medical Informatics & Decision Making* 2012, 12: 74 10.1186/1472-6947-12-74 22812565 PMC3444354

[37] International Organization for Standardization ISO IEC 62366–1: Medical devices—Part 1: Application of usability engineering to medical devices. Author: Geneva, Switzerland, 2015.

[38] Harte R.; Glynn L.; Rodríguez-Molinero A.; Baker P. M. A.; Scharf T.; Quinlan L.R.; et al A human-centered design methodology to enhance the usability, human factors, and user experience of connected health systems: A three-phase methodology. *JMIR Human Factors* 2017, 4(1): e8 10.2196/humanfactors.5443 28302594 PMC5374275

[39] Martin J.L.; Murphy E.; Crowe J.A.; Norris B.J. Capturing user requirements in medical device development: The role of ergonomics. *Physiological Measurement* 2006, 27, 49–62. 10.1088/0967-3334/27/8/R01 16772664

[40] Privitera M.B.; Evans M.; Southee D. Human factors in the design of medical devices–Approaches to meeting international standards in the European Union and USA. *Applied Ergonomics* 2017, 59, 251–263. 10.1016/j.apergo.2016.08.034 27890135

[41] Borsci S.; Uchegbu I.; Buckle P.; Ni Z.; Walne S.; Hanna G.B. Designing medical technology for resilience: Integrating health economics and human factors approaches. *Expert Review of Medical Devices* 2018, 15(1), 15–26. 10.1080/17434440.2018.1418661 29243500

[42] International Organization for Standardization. ISO 13407: Human-centered design processes for interactive systems. Author: Geneva, Switzerland, 1999.

[43] International Organization for Standardization. ISO 14155: Clinical investigation of medical devices for human subjects–Good clinical practices. Author: Geneva, Switzerland, 2011.

[44] International Organization for Standardization. ISO 14971: Medical devices–application of risk management to medical devices. Author: Geneva, Switzerland, 2012.

[45] Martin J.L.; Norris B.J.; Murphy E.; Crowe J. A. Medical device development: The challenge for ergonomics. *Applied Ergonomics* 2008, 39, 271–283. 10.1016/j.apergo.2007.10.002 18061139

[46] Vincent C.J.; Li Y.; Blandford A. Integration of human factors and ergonomics during the medical device design and development: It’s all about communication. *Applied Ergonomics* 2014, 45, 413–419. 10.1016/j.apergo.2013.05.009 23778022

[47] Bardin L. L’analyse de contenu. Quadrige/PUF: Paris, France, 2007.

[48] Strisland F.; Svagård I.S.; Austad H.O.; Reitan J. Meeting end user needs in collaborative medical device technology development research projects: A qualitative case study. *Studies in Health Technology and Informatics* 2017, 237, 49–54. 10.5334/ijic.2591 28479542

[49] Ciurana J. Designing, prototyping and manufacturing medical devices: An overview. *International Journal of Computer Integrated Manufacturing* 2014, 27(10), 901–918. 10.1080/0951192X.2014.934292

[50] Borsci S.; Macredie R.D.; Martin J.L.; Young T. How many testers are needed to assure the usability of medical devices? *Expert Reviews Medical Devices* 2014, 11(5), 513–525. 10.1586/17434440.2014.940312 25033757

[51] Borsci S.; Macredie R.D.; Barnett J.; Martin J.; Kuljis J.; Young T. Reviewing and extending the five-user assumption: a grounded procedure for interaction evaluation. *ACM Transactions on Computer-Human Interaction (TOCHI)* 2013, 20(5): 29.

[52] Quinn P. The EU commission’s risky choice for a non-risk based strategy on assessment of medical devices. *Computer Law & Security Review* 2017, 33, 361–370. 10.1016/j.clsr.2017.03.019

[53] Maresova P.; Penhaker M.; Selamat A.; Kuca K. The potential of medical device industry in technological and economical context. *Therapeutics and Clinical Risk Management* 2015, 11, 1505–1514. 10.2147/TCRM.S88574 26491337 PMC4599058

[54] Santos I.C.T.; Tavares J.M.R.S. Additional peculiarities of medical devices that should be considered in their development process. *Expert Reviews Medical Devices* 2013, 10(3), 411–420. 10.1586/erd.12.89 23668711

[55] Parreira P.; Sousa L.B.; Marques I.A.; Santos-Costa P.; Braga L.M.; Cruz A.; et al Double-chamber syringe versus classic syringes for peripheral intravenous drug administration and catheter flushing: a study protocol for a randomized controlled trial. *Trials* 2020, 21:78 10.1186/s13063-019-3887-1 31937342 PMC6961373

