# Supplementary material for: Development of an innovative double-chamber syringe for intravenous therapeutics and flushing: Nurses’ involvement through a human-centred approach
Source: PLoS One. 2020 Jun 25;15(6):e0235087. doi: 10.1371/journal.pone.0235087 (PMC7316231; doi:10.1371/journal.pone.0235087)
Supplement: S1 File — (PDF) [file pone.0235087.s001.pdf]

Painel de Peritos Expert Panel

Avaliação da usabilidade / ergonomia do protótipo semi-funcional da Seringa DUO

Usability/ergonomic assessment of the DUO Syringe semi-functional prototype

“Seringa DUO: Dispositivo inovador para administração endovenosa”

“DUO Syringe: Innovative device for intravenous administration”

(projeto POCI-01-0247-FEDER-017604)

1. Introdução Introduction

1.1 Contacto inicial com os participantes Initial contact with the participants

Os moderadores devem encontrar-se com todos os participantes num local e data/hora previamente definidos, devendo reservar alguns minutos para se apresentar e para agradecer aos participantes a sua presença. Os participantes poderão falar uns com os outros e/ou esclarecer alguma questão prévia com os moderadores, se necessário. Moderators from the research team should meet with all participants at a predefined place and date/time, and take a few minutes to introduce themselves and to thank participants for their presence. Participants will be able to talk to each other and/or clarify any previous questions with the moderators if necessary.

1.2 Obtenção de consentimento informado Informed consent

Apresentação da estrutura e conteúdo da sessão de discussão (grupo focal) e do modo como a informação irá ser recolhida e tratada. Presentation of the structure and content of the focus group, how information will be collected and treated.

> A sessão terá a duração de 1hora sensivelmente. Os participantes podem interromper e terminar a sua colaboração a qualquer momento, no caso de não desejarem continuar. A sessão será gravada em formato áudio para que não seja perdida nenhuma informação. A informação obtida será transcrita, mas não existirá, nessa transcrição, qualquer tipo de identificação pessoal. As gravações apenas serão ouvidas e transcritas pela equipa de investigação, sendo eliminadas assim que os trabalhos estiverem concluídos. Salvaguarda-se, em todo o processo, o anonimato e a confidencialidade dos participantes e dos dados. A informação obtida será utilizada exclusivamente para fins de investigação. The session will last approximately 1 hour. Participants may interrupt and finish their collaboration at any time. The session will be audio recorded. The information will be transcribed without any personal identification. Recordings will only be heard and transcribed by the research team and will be deleted as soon as the work is completed.

Throughout the process, the privacy and confidentiality of participants and data are maintained. The information obtained will be used solely for research purposes.

É solicitada a leitura e a assinatura do termo de consentimento informado e são esclarecidas todas as dúvidas e questões adicionais por parte dos participantes. Reading and signing the informed consent form is requested. All additional questions and concerns from the participants are clarified by the research team.

2. Apresentação do projeto Project presentation

Apresentação genérica do projeto Seringa DUO e, especificamente, do conceito do dispositivo médico. Generic presentation of the DUO Syringe project and the medical device concept.

O projeto "Seringa DUO: Dispositivo inovador para administração endovenosa" (projeto POCI-01-0247-FEDER-017604) visa o desenvolvimento de um novo dispositivo médico, nomeadamente de uma seringa sequencial de dupla câmara para administração de medicação por via endovenosa. Especificamente, esta seringa de dupla câmara (uma câmara para solução de lavagem e uma câmara para medicação) irá permitir a avaliação da permeabilidade do cateter, a administração de medicação e a subsequente lavagem do cateter. Assim, pretende-se que a seringa permita o carregamento *in situ* das duas câmaras (medicamento e solução de lavagem), de modo independente e, de seguida, a administração dos dois líquidos em quantidades iguais ou distintas. The project "DUO Syringe: Innovative device for intravenous administration" (project POCI-01-0247-FEDER-017604) aims to develop a new medical device: a sequential double chamber syringe for intravenous administration. Specifically, this dual chamber syringe (with one chamber for the flush solution and another chamber for medication) will allow for catheter permeability assessment, medication administration and subsequent flushing. Thus, it is intended that the syringe permits the in situ loading and administration of the two chambers (medication and flush solution) in equal or distinct amounts.

3. Apresentação dos conceitos para o dispositivo médico Medical device concepts presentation

Apresentação do vídeo elaborado pelo PIEP relativamente aos conceitos para o DM. Video presentation regarding the medical device concepts.

4. Apresentação dos protótipos semi-funcionais do dispositivo médico Medical device semi-functional prototypes presentation

Apresentação e distribuição dos protótipos semi-funcionais do dispositivo médico. Presentation of semi-functional prototypes of the medical device.

5. Avaliação Assessment

Nesta fase, pretende-se uma discussão integrada das características dos protótipos semi-funcionais da Seringa DUO, possibilitando que a conversa/troca de ideias decorra de modo espontâneo e livre. At this stage, an integrated discussion of the characteristics of the DUO Syringe semi-functional prototypes is intended, allowing the conversation/exchange of ideas to take place spontaneously and freely.

Se necessário, dirigir a discussão de modo a que sejam avaliados aspetos importantes da usabilidade e ergonomia do dispositivo médico, como por exemplo: If necessary, direct the discussion so that important aspects of medical device usability and ergonomics, such as:

- dimensões do corpo da seringa (syringe body dimensions);
- dimensões dos êmbolos (plunger dimensions);
- inferências acerca da possibilidade de erro na sequência das tarefas a realizar (inferences about the possibility of error regarding tasks to be performed);
- inferências acerca da facilidade de carregamento (inferences about ease of loading);
- inferências acerca da facilidade de administração (inferences about ease of administration);
- inferências acerca do trauma induzido no paciente com a utilização da Seringa DUO (inferences about patient-induced trauma with DUO Syringe).

Salientar que é um dispositivo médico que está a ser desenvolvido e, como tal, nesta fase, é de extrema importância uma análise minuciosa das suas características, considerando igualmente possíveis sugestões de alteração (que visem melhorias ao nível dos aspetos da usabilidade/ergonomia). Is importance to note that this medical device is being developed. As such, at this stage, a thorough analysis of its features is extremely important, also considering possible suggestions for changes (aimed at improvements in usability / ergonomics aspects).

6. Recolha de dados demográficos e encerramento Demographics collection and closure

É solicitado o preenchimento da ficha de dados sócio-demográficos a cada um dos participantes. Agradecer aos participantes o seu envolvimento no estudo. Each participant is required to complete the socio-demographic data sheet. Thank participants for their involvement in the study.

Ficha de caracterização Characterization sheet

ID ______

Idade: **Age**

_____ anos years

Género: **Sex**

- ▢ Masculino Male
- ▢ Feminino Female

Escolaridade: **Education**

- ▢ Bacharelato Bachelor
- ▢ Licenciatura Graduation
- ▢ Pós-graduação/Especialização Post-graduate/Specialty
- ▢ Mestrado Master degree
- ▢ Doutoramento PhD

Tempo de exercício profissional: ____

Professional duration

Hospital onde trabalha:

Hospital

- ▢ Público Public
- ▢ Privado Private
- ▢ Parceria público-privada Public/Private Partnership
- ▢ Outro Other (especificar specify ________________________)

Unidade/serviço onde trabalha:

Unit/Department

____

____

Há quanto tempo exerce funções nesta unidade/serviço? Unit/Department duration of professional exercise

____

- Muito obrigado pela sua colaboração –

- Thank you -
