# Supplementary figures and images for: Development of an innovative double-chamber syringe for intravenous therapeutics and flushing: Nurses’ involvement through a human-centred approach
Source: PLoS One. 2020 Jun 25;15(6):e0235087. doi: 10.1371/journal.pone.0235087 (PMC7316231; doi:10.1371/journal.pone.0235087)

**Design solution 1.**

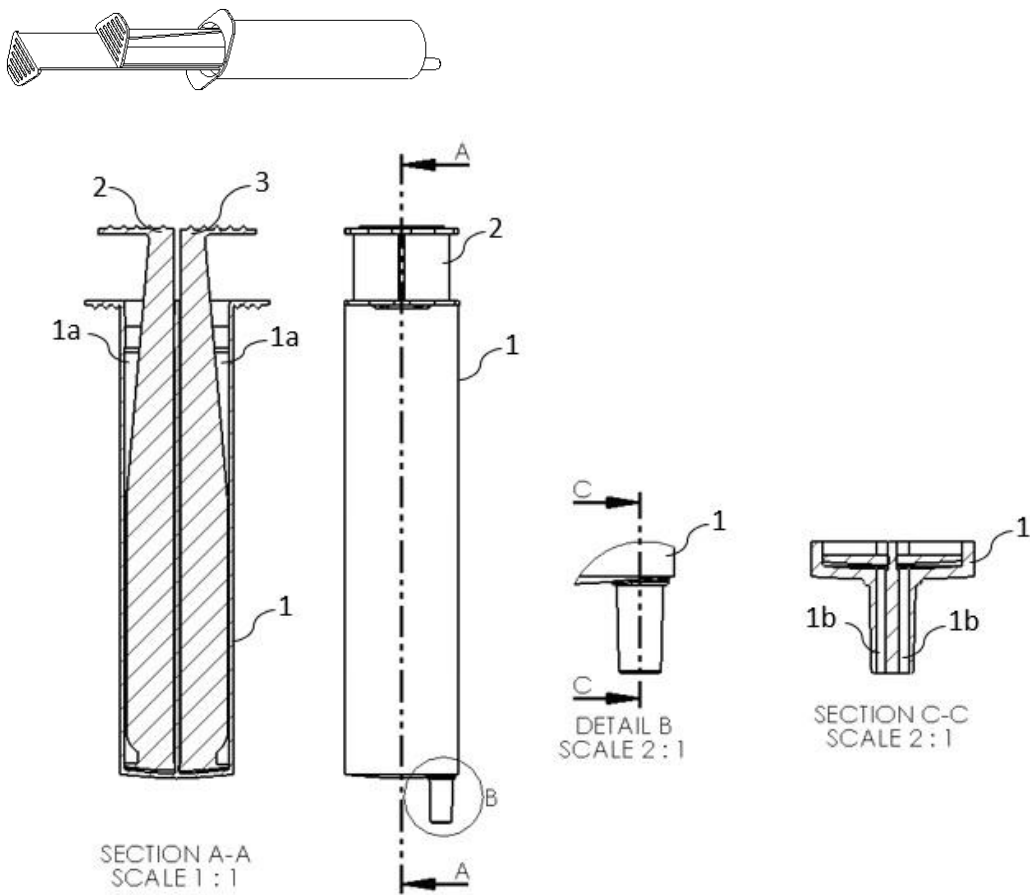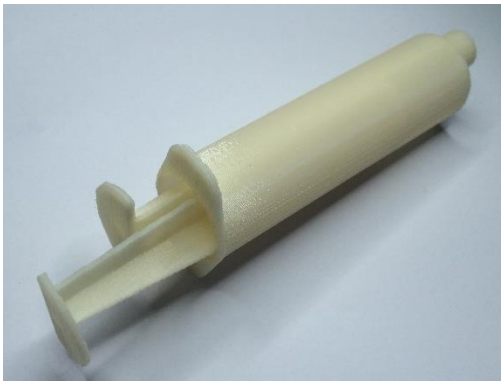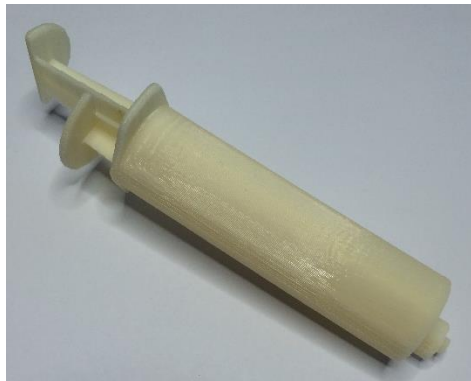

Design solution 3.

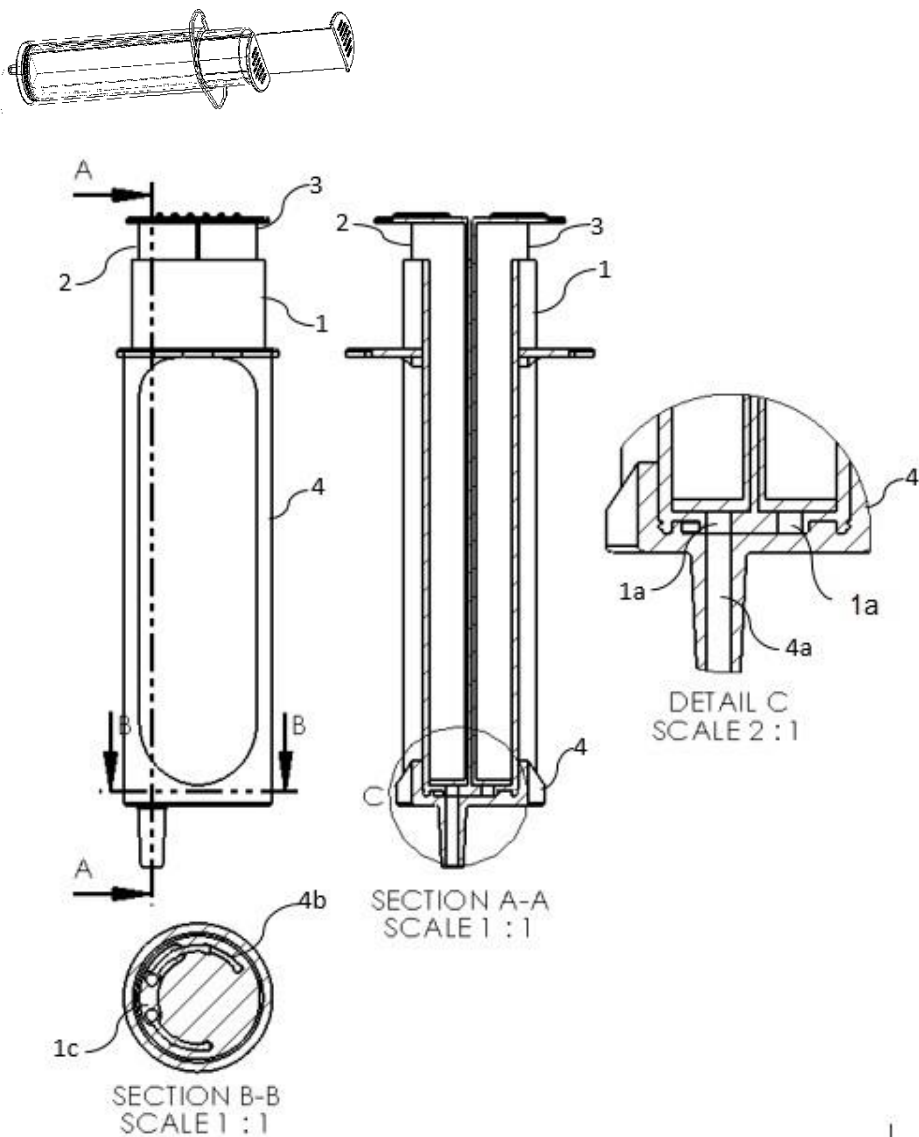

Design solution 4.

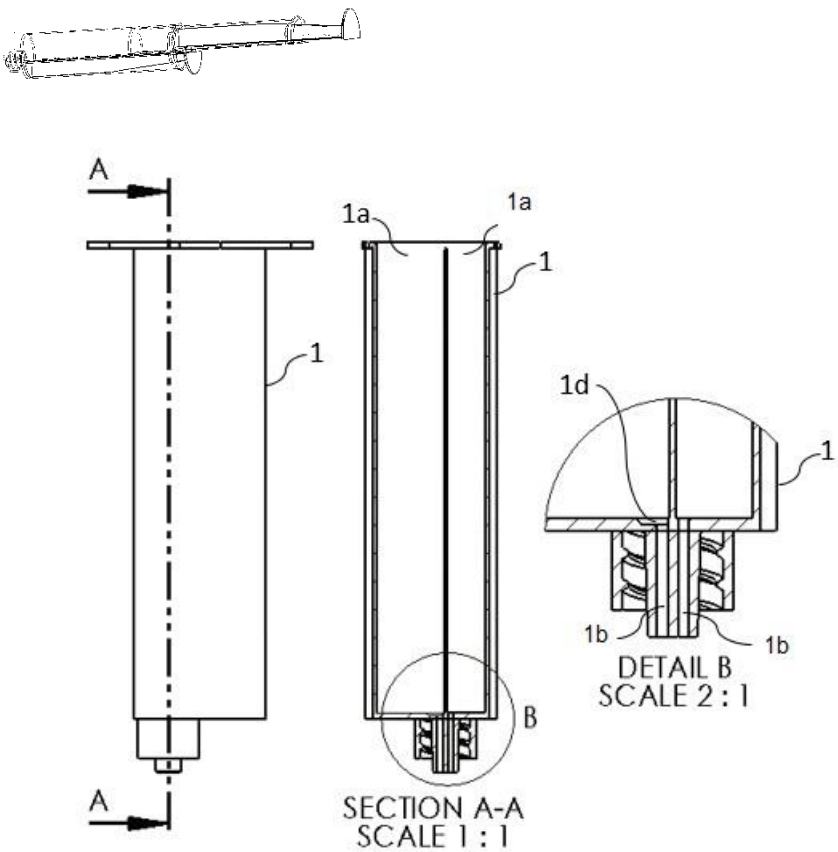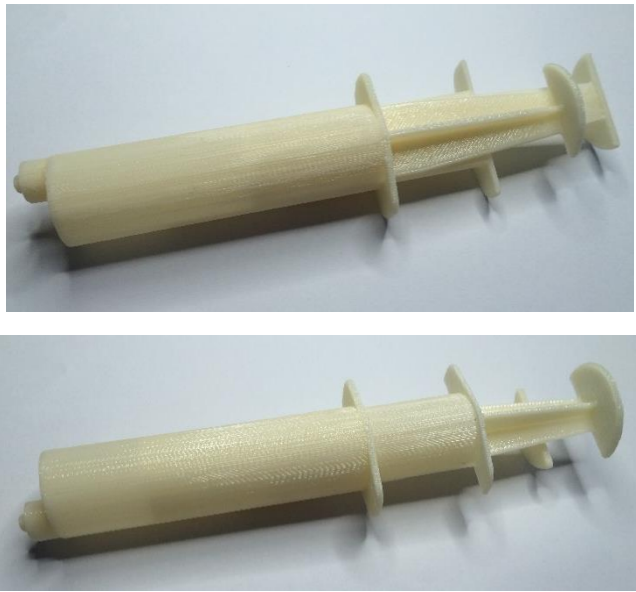

Design solution 5.

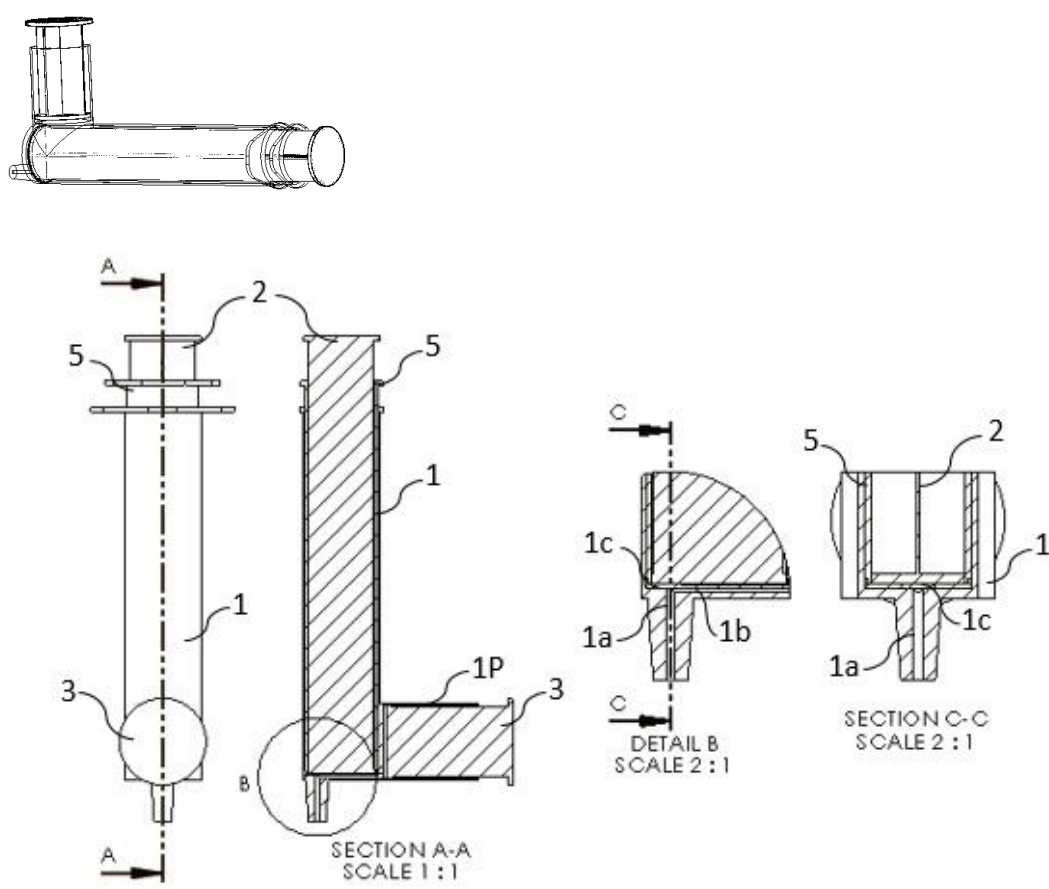

Design solution 6.

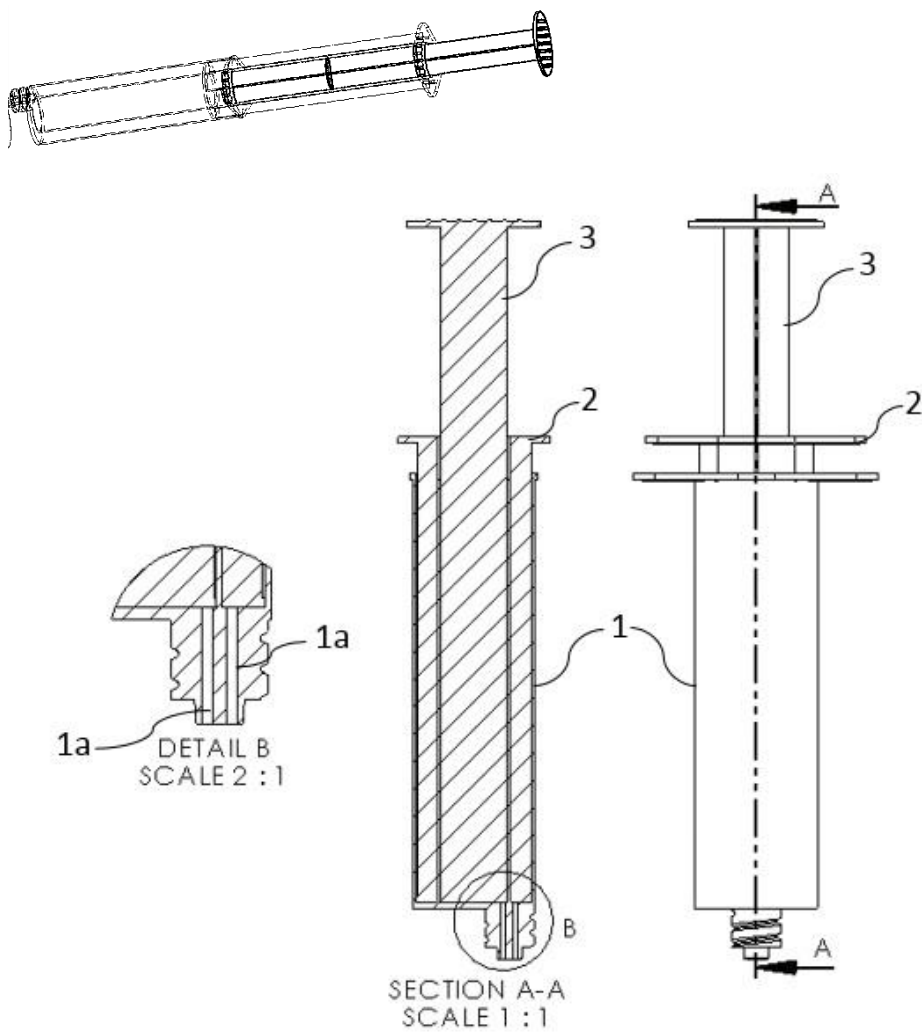

Supplement: S2 File — (PDF) [file pone.0235087.s002.pdf]
